# Blood-Cell-Based Inflammatory Markers as a Useful Tool for Early Diagnosis in Colorectal Cancer

**DOI:** 10.3389/fmed.2022.843074

**Published:** 2022-06-20

**Authors:** Maria Hernandez-Ainsa, Raul Velamazan, Angel Lanas, Patricia Carrera-Lasfuentes, Elena Piazuelo

**Affiliations:** ^1^Service of Digestive Diseases, University Clinic Hospital, Zaragoza, Spain; ^2^Aragón Health Research Institute (IIS Aragón), Zaragoza, Spain; ^3^Department of Medicine, Psychiatry and Dermatology, University of Zaragoza, Zaragoza, Spain; ^4^Biomedical Research Networking Center in Hepatic and Digestive Diseases (CIBERehd), Madrid, Spain; ^5^Aragón Health Sciences Institute (IACS), Zaragoza, Spain

**Keywords:** colorectal cancer, inflammation, neutrophil/lymphocyte ratio, platelet/lymphocyte ratio, systemic immune-inflammation index (SII), diagnosis

## Abstract

**Background:**

Systemic inflammation seems to be involved in the pathogenetic pathways of colorectal cancer (CRC). Analytical markers that reflect the inflammatory status, such as neutrophil/lymphocyte ratio (NLR), platelet/lymphocyte ratio (PLR) or systemic immune-inflammation index (SII), have been proposed as tools for the prognosis of CRC. Nevertheless, their use for diagnosis has been scarcely investigated.

**Aims:**

To analyze the ability of these markers and of a new marker combining SII and hemoglobin concentration, named NP/LHb = [neutrophils x platelets]/[lymphocytes x hemoglobin], as tools for CRC diagnosis. Furthermore, we studied their association with CRC-related variables.

**Methods:**

Case-control study including 214 CRC patients and 214 controls without CRC, matched by age (±5 years) and sex. We collected demographic, CRC-related and laboratory variables to calculate NLR, PLR, SII, and NP/LHb. In the case group, the laboratory variables were collected at two different period times, 6 months (IQR 4–8) before the CRC diagnosis and at the time of the diagnosis. ROC analysis was performed to evaluate the discriminatory accuracy of each index and we calculated Se, Sp, PPV, NPV, and OR to identify the diagnostic performance of each positive marker.

**Results:**

NP/LHb showed high Sp (92.06%) and PPV (87.50%) to diagnose patients with CRC. This index exhibited an OR of 14.52 (8.26–25.52) and the best area under the curve (AUC: 0.78) for a positive CRC diagnosis. We found significant differences in all indices according to the presence of CRC, observing the highest values in CRC patients at time of diagnosis, in comparison with the analysis performed in the previous months to diagnosis or with control patients. There were significant differences in all ratios according to TNM stages (*p* < 0.05). PLR, SII and NP/LHb (but not NLR) showed significant differences according to tumor location (*p* < 0.05). Right-sided colon cancers presented the highest values, in comparison with left-sided and rectal cancers.

**Conclusions:**

Systemic inflammatory cell ratios (especially NP/LHb) change over time with the development of CRC, so they could be useful in its early diagnosis. We suggest that they could be routinely measured in patients with suspicion of CRC, to identify those ones with a higher risk of cancer, considering the high positive predictive value they have shown in our study.

## Introduction

Colorectal cancer (CRC) is the most common cancer in western societies when men and women are considered together and its incidence is increasing (1, 2). Stabilizing and decreasing CRC mortality is an essential objective worldwide. Most developed countries have programs (3) for the early detection of CRC, mostly based on fecal occult blood test (FOBT) and colonoscopy (4). However, these strategies sometimes fail to diagnose CRCs due to different factors such as the ocurrence of interval colorectal cancer after a negative colonoscopy, the low participation rates in these programs, or, less frequently, a false negative result. Also, colonoscopy is an invasive test that can cause complications and the false-positive rate of the FOBT cannot be ignored, resulting in the performance of colonoscopies in patients without pathology on many occasions (4), a situation that can lead to an overload of endoscopy departments, a very unfavorable condition considering the current health situation. These facts suggest the need to look for new tests or biomarkers, in order to improve the early diagnosis of CRC in these patients and improve the accuracy of existing tests.

The pathogenesis of CRC seems to be driven by systemic inflammation (5–8), in addition to the molecular pathways already known (9). The persistence of intrinsic inflammation, produced by the tumor environment, together with the extrinsic inflammation, which is favored by the pro-inflammatory state of the organism, lead to an increase in the tumor cell proliferation, angiogenesis and inhibition of apoptosis (5). Therefore, the interaction between the processes of systemic inflammation and the host's immune response would be involved in the initiation, development and progression of the tumor (10). Cancer-related inflammation is represented by complex mechanisms that include pro-inflammatory cytokines, growth factors, immune cells, and other proteins (5, 6). The balance between all these inflammatory mediators would be analytically reflected by the blood levels of platelets and leukocytes, especially neutrophils and lymphocytes (11, 12). Recently, several studies in this area have suggested that the use of blood analytical markers, composed by the combination of systemic inflammation parameters, could provide useful information regarding the prognosis of CRC (13–15), as well as other cancerous (16–18) or inflammatory processes. Some of the proposed biomarkers are platelet/lymphocyte ratio (PRL) (17, 19–21) and neutrophil/lymphocyte ratio (NLR) (16, 22–26). Another proposed marker is SII or systemic immune-inflammation index, calculated by using the formula SII = (P x N) / L, where P, N, and L correspond to platelets, neutrophils, and lymphocytes, respectively (18, 27–29).

It has been observed an association between higher levels of the inflammatory markers and the risk of CRC progression, which could traduce in a worse prognosis (10, 27). Moreover, some studies in the recent years have reported significant differences between right-sided colon cancers (RCC) and left-sided colon cancers (LCC), according to the inflammatory ratios values, with different survival and prognosis due to them (30, 31). In fact, an increasing evidence of the potential usefulness of these markers for prognostic prediction of several cancers such as gastric, lung, ovarian or pancreatic, has been reported in the last years (32–36), but their capacity as tools for cancer diagnosis has been barely investigated. The diagnostic efficacy of these indices in CRC remains unknown, and only a few studies have been reported (10, 37, 38).

The investigation of the sequential cell-ratios in people with a normal colonoscopy and people with CRC would help to establish their potential and usefulness as biomarkers for the diagnosis of CRC. Furthermore, it would be desirable to investigate the potential increase of their diagnostic yield by adding other analytical parameters related to the presence of a colorectal tumor, such as hemoglobin concentration (Hb). Hb would reflect the degree of anemia in these patients. Anemia due to a cancer status is usually multifactorial (gastrointestinal losses, inflammatory processes, other comorbidities associated, etc.). Based on this hypothesis, we formulated a new index named NP/LHb, not previously used, including hemoglobin concentration.

To summarize, we hypothesized that these blood-cell-based ratios could be not only a precise tool to assess the prognosis and the characterization of CRC, but also as an useful tool for the early diagnosis of CRC that could be used, alone or together with exiting tests, to increase the yield of current CRC prevention programs. Our main aim was to analyze the diagnostic value of these analytical markers and the new marker named NP/LHb, by exploring their ability to discriminate patients with and without CRC. Also, we evaluated whether there were differences between the marker's values at the time of cancer diagnosis and months before, in CRC patients. Furthermore, we explored the association between them and the different clinicopathological features of the tumor, such as the TNM stage and tumor location.

## Materials and Methods

### Study Population

In this retrospective case-control study, we included 214 patients with CRC (obtained from the registry of CRC diagnosed cases in our center), and 214 control patients without CRC diagnosis (obtained from the CRC screening program database of our center), between 2010 and 2018. Controls were chosen for each included case, adjusted by age (±5 years) and sex, and taking into consideration the inclusion and exclusion criteria showed below. This study was reviewed and approved by the Regional Ethical Committee of Aragón (CEICA). Written informed consent from the participants was not required since this study is retrospective and used data stored in databases, which were anonymized for data analysis.

The inclusion criteria for CRC patients were as follows: (1) The patient had a primary colorectal cancer confirmed by histopathology; (2) The patient had a blood test performed at the time of the CRC diagnosis; and (3) This blood test must have been performed before any type of therapeutic intervention (surgery, chemotherapy and/or radiotherapy) and in a baseline situation (excluding blood tests performed in a situation which could alter the total blood cell count, as an acute infection). The exclusion criteria were: (1) A diagnosis of *in situ* colorectal carcinoma; (2) A diagnosis of a systemic inflammatory process, an hematological disease or being under a pharmacological treatment that could modify the blood cell count; and (3) Not having an available blood test at the time of the CRC diagnosis that follow the exposed criteria.

The inclusion criteria for control patients were as follows: (1) They had to be included within the CRC screening population at our center and have a normal colonoscopy; (2) They should not have a personal or familiar history of CRC; and (3) Have a blood test within the previous year to the screening colonoscopy and performed in a baseline condition (excluding blood test performed in a situation which could alter the total blood cell count as an acute infection). The exclusion criteria for controls were: (1) Current or past diagnosis of another cancer; (2) Diagnosis of a systemic inflammatory process, an hematological disease or pharmacological treatment that could modify the blood cell count; and (3) Not having any available blood test within the previous year to the screening colonoscopy that follow the exposed criteria.

### Data Collection

The following inflammatory cell ratios were considered the main variables of the study:


$$\begin{array}{l} NLR\;\left(neutrophil-to-lymphocyte\;ratio\right)\\ =\frac{Absolute\;number\;of\;neutrophils}{Absolute\;number\;of\;lymphocytes}\\ PLR\;\left(platetets-to-lymphocyte\;ratio\right)\\ =\frac{Absolute\;number\;of\;platelets}{Absolute\;number\;of\;lymphocytes} \end{array}$$

$$\begin{array}{l} SII\;(systemic\;immune-inflammation\;index)=\;\frac{\left[\left(Absolute\;number\;of\;neutrophils\right)x\;\left(Absolute\;number\;of\;platelets\right)\right]}{Absolute\;number\;of\;lymphocytes}\\ \;\frac{NP}{LHb}ratio\;=\;\frac{\left[\left(Absolute\;number\;of\;neutrophils\right)x\;\left(Absolute\;number\;of\;platelets\right)\right]}{\left[\left(Absolute\;number\;of\;lymphocytes\right)x\;\left(Haemoglobin\;g/dl\right)\right]} \end{array}$$

In the CRC group, these laboratory variables were collected at two different period times. Due to this, the blood cell ratios were calculated twice. One determination was from the blood test performed at the time of CRC diagnosis. The other one was calculated from a blood test performed before the diagnosis (median 6 months, IQR 4–8). Having a blood test at the time of diagnosis was one of the requirements to be included in the case group. However, it was not mandatory to be included to have an analysis from a period prior to diagnosis. In our case group (*n* = 214 patients), 131 were those who had a blood test previously to be diagnosed. The maximum and minimum period marked, in which this blood test was performed, was 12 and 3 months before the CRC diagnosis, respectively.

In the control group, we calculated the ratios only once based on the analytical variables collected from the blood test performed nearer to the screening colonoscopy (median 3 months, IQR 1–6).

Furthermore, we collect demographic variables (age and sex) for both groups and the following CRC-related variables in the case group: tumor-nodes-metastases (TNM) stage, tumor invasion (T), lymph nodes (N), metastases (M), tumor location, differentiation level and symptoms at diagnosis. The symptoms collected were: rectal bleeding, change in bowel habit, anemia, abdominal pain, constitutional syndrome and obstruction. Constitutional syndrome is the clinical entity that include fatigue, anorexia and involuntary weight loss.

### Statistical Analysis

An initial descriptive exploratory analysis of all clinical variables was carried out. Qualitative variables were expressed as frequencies and percentages, whereas continuous variables were reported as mean with standard deviation (SD) or median with interquartile range (IQR). Normality was tested using the Shapiro-Wilk test.

Differences between independent groups were evaluated with Chi-square (χ2) test for qualitative variables and with Mann-Whitney or Kruskal-Wallis test for continuous variables. Wilcoxon test was performed to evaluate the same analytical index at different times.

The discriminatory accuracy of each analytical marker to diagnose CRC was evaluated using the area under the receiver operating characteristics (ROC) curve (AUC). DeLong test was used to test the statistical significance of the difference between the areas under two dependent ROC curves. The Youden index was used to identify the optimal cut-off value to discriminate CCR. In order to evaluate the diagnostic performance of each positive marker, sensitivity (Se), specificity (Sp), positive predictive value (PPV), negative predictive value (NPV) and odds ratio (OR) with 95% confidence intervals (CI) were calculated.

The level of significance in the study was established at 0.05. Data was analyzed using the Jamovi software for iOS, version 1.6.15.

## Results

A total of 428 patients were finally included in the study. No statistical difference in age and sex were found between cases and controls. The mean age in the CRC group was 67.08 ± 11.08 and 65.60 ± 3.09 years in the control group. The proportion of males/females was the same in both groups (140/74). The baseline characteristics of CRC patients (reflected by tumor-related variables collected) are shown in Table 1.

**Table 1:** Clinical characteristics of CRC patients.

	**CRC patients (*****n*** **=** **214)**	
**CRC-related variables**	***n***	**%**
**TNM stage**		
I	36	16.82%
II	51	23.83%
III	81	37.85%
IV	46	21.50%
**Location**		
Rectal	56	26.17%
Left colon	84	39.25%
Right colon	73	34.11%
Not available	1	0.47%
**Symptoms at diagnosis**		
Rectal bleeding	37	17.29%
Change in bowel habit	33	15.42%
Anemia	33	15.42%
Abdominal pain	28	13.08%
Constitutional syndrome	15	7.01%
Obstruction	14	6.54%
Other	22	10.28%
Not available	32	14.95%
**Differentiation level**		
G1	37	17.29%
G2	125	58.41%
G3	32	14.95%
Not available	20	9.35%

*CRC, colorectal cancer; TNM stage, tumor-nodes-metastases stage; n, total number of patients; %, frequency of patients*.

### NLR, PLR, SII and NP/LHB Indexes in CRC and Controls

**Figure 1:**
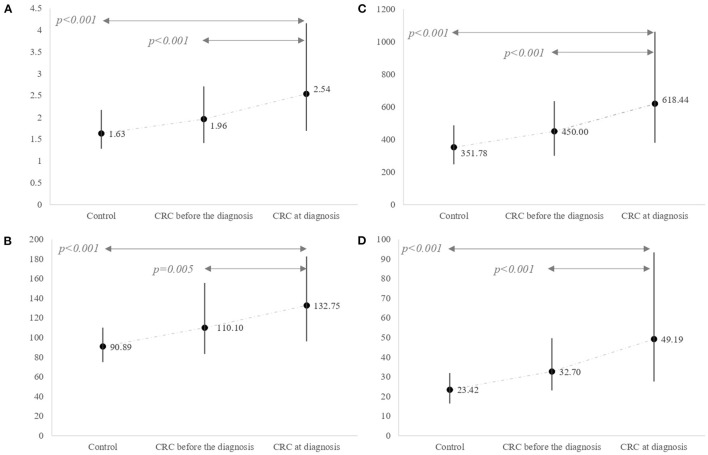
Differences in inflammatory marker levels between the control group and the CRC group. The CRC group was classified according to two different period times. One refers to markers estimated based on blood tests performed several months (median 6 months, IQR 4–8) before the CRC diagnosis and the other one at the diagnosis index time. **(A)** NLR (median and IQR); **(B)** PLR (median and IQR); **(C)** SII (median and IQR); **(D)** NP/LHb (median and IQR). NLR, neutrophil-to-lymphocyte ratio; PLR, platelet-to-lymphocyte ratio; SII, systemic immune-inflammation index; NP/LHb, neutrophils x platelets/lymphocytes x hemoglobin ratio.

As shown in Figure 1, the levels of NLR, PLR, SII, and NP/LHb were significantly increased in CRC patients, compared to control patients. Specifically, CRC patients showed the highest values of the markers at the moment of the diagnosis, in comparison with their previous values obtained in a pre-diagnosis analysis *(p*< *0.05)* and with the control group of patients without CRC *(p*< *0.05)*.

### Association of the Blood Analytical Markers According to CRC-Related Variables

Levels of NLR, PLR, SII, and NP/LHb were significantly associated with TNM stage (Table 2). The tumor stages were classified according to the 8th edition of the American Joint Committee on Cancer (AJCC) cancer staging system. The more advanced the TNM stage was, the higher value presented the markers. Comparison between early TNM stage (I) and more advanced TNM stages (II, III and IV) showed significant differences in the four indices. Nevertheless, no significant difference was observed when compared between II, III and IV TNM stages. According to the tumor location, PLR, SII and NP/LHb showed significant association with this variable, but no significant relation was identified between NLR and tumor location (Table 2). Patients with right-sided colon cancer (RCC) had significantly higher values compared to left-sided colon cancer (LCC) and rectal cancer (RC) patients. We did not find association between TNM stage and tumor location (*p* = *0.866*).

**Table 2:** Median, interquartile range (IQR) and association of the inflammatory markers according to CRC-related variables (TNM stage and tumor location).

	**NLR median (IQR)**	**PLR median (IQR)**	**SII median (IQR)**	**NP/LHb median (IQR)**
**TNM stage**				
**I (*****n*** **=** **36)**	1.88 (1.36–2.36)**	103.96 (83.32–133.59)*	419.70 (336.74–628.54)**	28.69 (23.71–57.41)**
**II (*****n*** **=** **51)**	3.16 (1.86–5.92)	122.86 (88.08–194.00)	708.40 (312.41–1403.45)	57.90 (27.40–130.77)
**III (*****n*** **=** **81)**	2.79 (1.71–4.22)	139.47 (102.57–178.60)	637.78 (397.96–1001.30)	49.32 (28.20–84.64)
**IV (*****n*** **=** **46)**	2.64 (1.78–4.18)	157.31 (97.34–216.09)	814.00 (424.60–1208.51)	66.47 (33.41–107.25)
*p-value*	*** <0.001***	***0.011***	***0.034***	***0.020***
**Tumor location**				
**Rectal (*****n*** **=** **56)**	2.27 (1.69–3.41)	111.89 (87.27–156.52)	495.97 (339.85–835.45)	35.15 (24.21–61.18)
**Left colon (*****n*** **=** **84)**	2.56 (1.44–4.07)	133.73 (96.60–182.88)	654.13 (391.28–1275.89)	48.66 (27.34–96.87)
**Right colon (*****n*** **=** **73)**	3.25 (1.85–4.79)	158.82 (103.78–205.78)	724.14 (430.27–1245.74)	66.78 (31.01–121.54)
*p-value*	*0.055*	***0.031***	***0.007***	***0.001***

NLR, neutrophil/lymphocyte ratio; PLR, platelet/lymphocyte ratio; SII, systemic immune-inflammation index; NP/LHb, neutrophils x platelets/lymphocytes x hemoglobin ratio; IQR, interquartile range; TNM stage, tumor-nodes-metastases stage. Asterisk (^*^) indicates a significant difference (p < 0.05) between groups (I vs. III, IV). Two asterisks (^**^) show a significant difference (p < 0.05) between groups (I vs. II, III, IV). Bold P-values indicates p < 0.05.

In addition to TNM stage and tumor location, we analyzed other CRC-related variables. Significant differences were found between tumor invasion (T) and NLR, PLR, SII and NP/LHb, with *p* = *0.003, p* = *0.047, p* = *0.007* and *p* = *0.011*, respectively. However, no statically significant differences were found for lymph nodes (N), metastases (M) and tumor differentiation variables (Supplementary Table 1). Finally, we observed differences according to the symptoms at diagnosis, specifically with constitutional syndrome and intestinal obstruction. These two symptomatic presentations showed significant higher markers values, in comparison with other symptoms at diagnosis such as rectal bleeding, anemia, change in bowel habit or abdominal pain (Supplementary Table 2). Moreover, we identified a significant association between patients who presented constitutional syndrome and NLR (*p* = *0.023)* and NP/LHb (*p* = *0.034)*, and also, a significant association was found between patients who presented intestinal obstruction and NLR (*p* < *0.001*), PLR (*p* = *0.015*), SII (*p* = 0.000) and NP/LHb (*p* < *0.001*).

### Evaluation of the Diagnostic Efficacy of the Blood Analytical Markers

**Figure 2:**
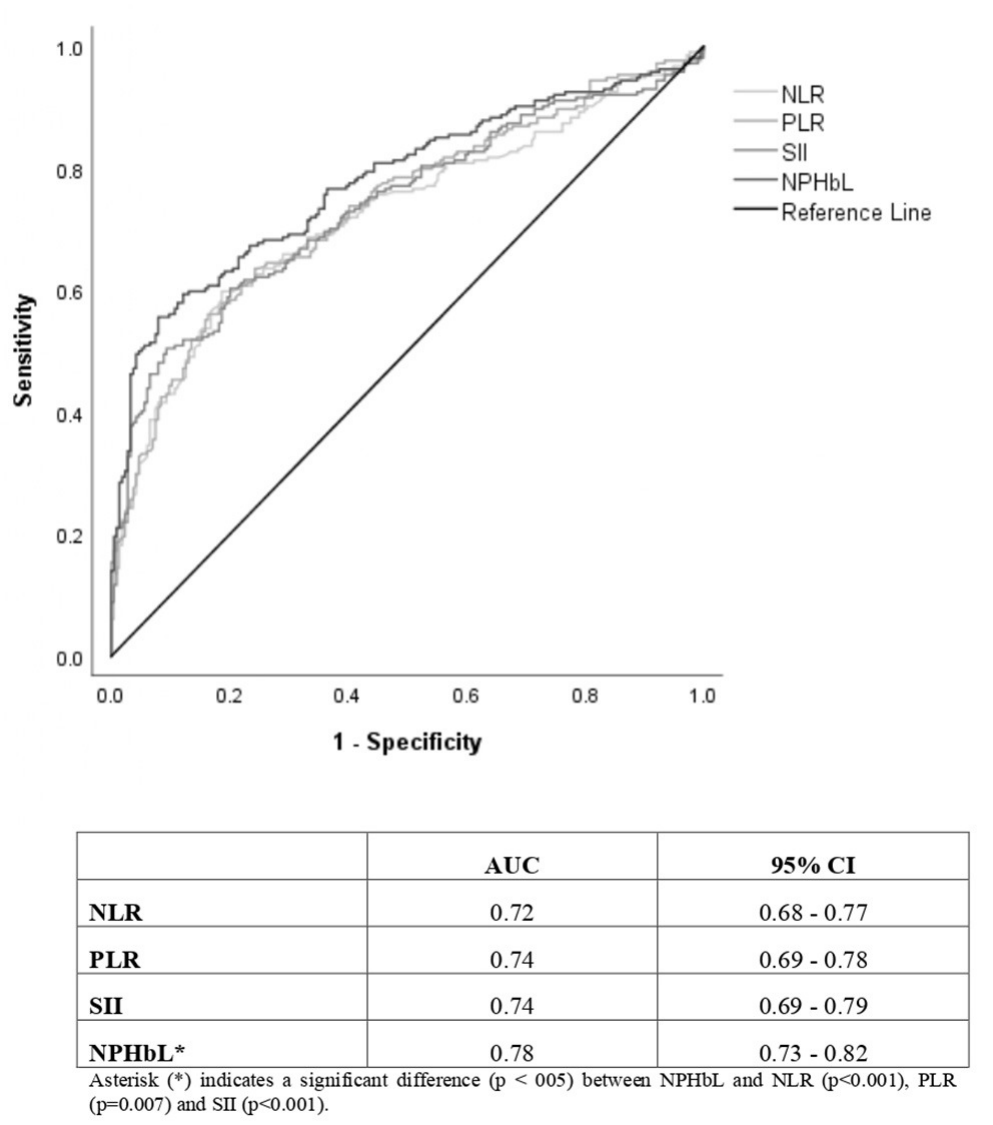
ROC (receiver-operating characteristic) curves and area under the curve (AUC) with confidence interval (95% CI) of the inflammatory markers based on CRC diagnosis as the outcome variable. NLR, neutrophil-to-lymphocyte ratio; PLR, platelet-to-lymphocyte ratio; SII, systemic immune-inflammation index; NP/LHb, neutrophils x platelets/lymphocytes x hemoglobin ratio; CI, confidence interval.

The NP/LHb ratio showed significantly the best diagnostic value, with an AUC (95% CI) of 0.78 (0.73–0.82), in comparison with the other three analyzed ratios, with an AUC of 0.72 (0.68–0.77) for NRL, 0.74 (0.69–0.78) for PLR and 0.74 (0.69–0.79) for SII. These data are represented in Figure 2. Results of ROC (receiver-operating characteristic) curve analysis showed the optimal cut-off values and other diagnostic parameters of the four inflammatory markers, which are summarized in Table 3.

**Table 3:** Optimal cut-off values to discriminate CRC and other diagnostic parameters of the inflammatory markers.

**Markers**	**Cut-off**	**TP**	**FP**	**FN**	**TN**	**Se (%)**	**Sp (%)**	**PPV (%)**	**NPV (%)**	**OR (95% CI)**
**NLR**	2.28	128	40	86	174	59.81	81.31	76.19	66.92	6.47 (4.17–10.04)
**PLR**	122.40	120	35	94	179	56.07	83.64	77.42	65.57	6.53 (4.16–10.26)
**SII**	616.50	108	20	106	194	50.47	90.65	84.38	64.67	9.88 (5.80–16.84)
**NP/LHb**	43.90	119	17	95	197	55.61	92.06	87.50	67.47	14.52 (8.26–25.52)

*NLR, neutrophil/lymphocyte ratio; PLR, platelet/lymphocyte ratio; SII, systemic immune-inflammation index; NP/LHb, neutrophils x platelets/lymphocytes x hemoglobin ratio; TP, true positive; FP, false positive; FN, false negative; TN, true negative; Se, sensitivity; Sp, specificity; PPV, positive predictive value; NPV, negative predictive value; OR, odds ratio; CI, confidence interval*.

Furthermore, we found that the number of positive markers (this means a higher or equal value according to the cut-off point) calculated for each patient, significantly increases the risk of CRC, with an OR (95% CI): 1.60 (0.86–3.00), 2.73 (1.23–6.07), 7.37 (3.30–16.49) and 28.77 (12.63–65.54) for one, two, three or all positive markers respectively.

## Discussion

The implication of the systemic inflammation in the CRC pathogenesis has been studied in detail (5, 6). Within the tumor-induced systemic environment, numerous inflammatory cytokines are involved in colorectal cancer cell invasion and development of metastases (7, 8). The blood-cell-inflammation markers are ratios that link the different proportion of cells involved in the inflammatory status. Lymphocytes have a major anti-tumor role, participating in the cytotoxic cell death of tumor cells and in the production of cytokines that inhibit proliferation and metastatic spread (10). In contrast, neutrophils have a pro-tumorigenic effect, being the main source of cytokines, growth factors and proteases that regulate the tumor angiogenesis (10). Platelets are the major vascular endothelial growth factor (VEGF) transporter, and this growth factor promotes the formation of blood vessels in the tumor environment (11, 12, 39, 40), promoting the formation of metastasis. Considering this, the indices could reflect the pro/antitumorigenic inflammatory status in these patients.

In the present study, CRC patients showed higher values of these inflammatory indices and their values significantly differ from the values of patients without CRC, which is in concordance with previously published data (10, 37, 38, 41, 42). In addition to, and to our knowledge, this is the first study that also compare these inflammatory markers in CRC population at two different time intervals, presenting a clear increase in the index values with time. Furthermore, a new marker named NP/LHb has been tested for the first time, and it shows to be the best diagnostic index in comparison with the others inflammatory markers analyzed. The study of these indices in CRC asymptomatic patients (months before they become symptomatic and are diagnosed of CRC) and in control patients without colorectal cancer with a normal colonoscopy, provides valuable information, reinforcing the hypothesis of the progressive increase of these indices as the result of the systemic inflammatory process developed in the tumor microenvironment.

In our study, we have observed an increasing trend in the values of all indices studied during the time period where patients go from being asymptomatic to develop symptoms who led to a diagnosis of CRC. This evolution could reflect their dynamic changes on their way to CRC progression. The definition of the role of these inflammatory indices and the precise cut-off values before the cancer is diagnosed with current tests would identify high-risk populations and improve our screening methods, based on these complementary biomarkers.

Different studies have shown that high NLR, PLR, or SII values are associated with advanced TNM stage, more tumor invasion, poorer differentiation level and worse overall survival (OS) (10, 16–29). According to the findings of our study, NLR, PLR, SII and the new marker named NP/LHb were significantly associated with the TNM stage, showing an increasing value at a higher TNM stage. In fact, it has been reported that the total number of lymphocytes in patients diagnosed with colorectal cancer could be an independent prognostic factor and that a higher lymphocyte count at diagnosis could be related to a better prognosis of CRC in early stages (43). It has also been observed that patients with rectal cancer and higher levels of NLR have worse survival rates, even in early stages (I-II) (44). Based on this data, we analyzed the differences between TNM stages. We observed significant differences between early TNM stages (I) and advanced TNM stages (II-III-IV) in the four indices studied. However, we did not find significant differences between stages II, III and IV. Therefore, it suggests that these indices may have a good discriminative potential, especially when comparing early stages with more advanced stages, but they show worse results to discriminate between similar stages.

In relation to the association of the blood analytical markers according to the three variables that consists the TNM stage in a separated analysis (T = tumor invasion, N = lymph nodes and M = metastases), we obtained similar results according to those presented in other studies, especially related to T, in which articles they found significant association with T and the four blood studied markers, as we found too. However, and as it has been shown in other articles (42), it could not be demonstrated significant association between indexes and lymph nodes or metastases.

According to the tumor location, we found that PLR, SII and NP/LHb were significantly associated with this variable, but we did not find association for NLR. The higher values of the markers were found in patients with right-sided colon cancer (RCC), which is also in concordance with other studies, that found higher values of NLR and PLR in patients with RCC (30, 31), suggesting a prognostic value of these indices according to the tumor location. Moreover, in our study, we analyzed the relation between tumor location and TNM stage. We wanted to asses if the high values of the ratios observed in RCC were not because of the fact that there were more advanced TNM stages concentrated in this right location. We did not find any significant association between these two variables, and we found very similar frequencies of the four TNM stages in the three different tumor locations (right colon, left colon and rectal), which presume that the higher index values in RCC are independent from TNM stage in our population. The exact mechanisms that explain these significant differences are still unknown. It has been shown that molecular, biological, anatomical and clinical differences exist between RCC and LCC. There is evidence that RCC seem to be more aggressive and have a worse prognosis and survival, in comparison with left-sided colon cancers (LCC) (9, 45). Some authors have showed that right-sided colon may contain a more complex lymphatic system, which may more easily cause a cancer-related inflammatory response in RCC (31). In relation with this, some studies performed in colorectal tumor tissue samples have observed a more intense inflammatory response in the growth and development of RCC than in LCC (46). This interesting fact could be extrapolated to our results, presuming that the higher values of inflammatory ratios obtained in RCC could be due to a powerful inflammatory response of this cancer location in a systemic level. Other studies reported that patients with RCC tended to be older, showed deeper cancer infiltration and their diagnosis may be delayed compared to LCC diagnosis, perhaps due to more unspecific symptoms (47, 48). According to the clinical presentation, anemia is usually found in RCC, and rectal bleeding or change in bowel habit is more frequently observed in LCC (49). In addition to, other trials have reported that FOBT present lower sensitivity and specificity for diagnose RCC, obtaining a higher detection rate of left-sided colon and rectal cancers than that of left-sided cancers (50). In relation with this finding, we believe that the application of these indices could be especially useful in cancers developed in this right location.

We have found another interesting data related to symptoms at diagnosis and their association with the inflammatory markers. Patients with constitutional syndrome and intestinal obstruction at diagnosis presented the higher values of the inflammatory indices in comparison with patients that had anemia, abdominal pain or rectal bleeding at diagnosis. Nevertheless, only intestinal obstruction showed significant association with the four indices. These findings could be related to the fact that patients who present with an obstruction usually have advanced tumors.

To evaluate the potential diagnostic use of these ratios in colorectal cancer, firstly we examined their optimal cut-off points, which were found after ROC analysis. Although NP/LHb ratio was the marker that obtained the best AUC, all of them had very similar AUC values. In some studies, it has already been published that the combination of different indices shows an increase in the accuracy to discriminate CRC (38, 41, 42). Despite the fact that all the markers provided low sensitivities (<60%) and low negative predictive values (<70%), they provided high specificities (>80%) and positive predictive values (>70%). In particular, the new marker NP/LHb showed the best results in that field, with the highest specificity and positive predictive value. This means that in case of obtaining a positive result with this index (a value equal or higher than its established cut-off), the probabilities of having a CRC would be high, since the percentage of false positives would be low. Besides, the data suggests that the index will correctly classify more than 90% of patients as healthy patients when the value is below the established cut-off. Other finding that enhances the diagnostic utility of the new index is that it showed the highest OR in comparison with the others indices studied. Patients with this positive marker have 14.52 times greater risk of suffering from CRC than a patient with negative marker. Finally, our results showed that the more positive markers a patient have, the higher the probability of suffering from CRC it has.

Currently, the most important and developed CRC screening method in most country is the fecal occult blood test (FOBT). This method has an acceptable sensitivity and a good negative predictive value. However, and for this reason, there are a high number of non-pathological colonoscopies in patients with positive FOBT (around 40%), producing a non-negligible rate of false positives, with the extra cost and possible risks that such examinations entail. Our studied indices have obtained diagnostic parameters contrary to FOBT, with limited sensitivity and negative predictive value. We understand that they would not be good screening tests when used in isolation and as single tests, however, since they have a high positive predictive value and specificity, they could be good tools to be used together with other tests (e.g., FOBT) to ensure an accurate diagnosis and increase the probability for a positive diagnosis of CRC.

Therefore, the hypothesis that can be deduced from this work is that in the CRC screening population a FOBT (test with high sensitivity), can be performed first and then a blood test to determine the indices here reported (more specific test). This should prioritize patients with a higher risk of CRC for colonoscopy or other tests. The priority could be given to those patients who presented higher scores in NLR, PLR, SII and, specially, in NP/LHb. Another aspect in favor of the use of these indices is their low cost, simplicity, accessibility and easy interpretation, since they can be obtained directly from the hemogram in a blood extraction.

We are aware of the limitations of our study. It is a single-center and a retrospective study. In addition to, all analyzed markers may reflect a non-specific inflammatory response due to CRC or other tumors. For these reasons, further research in prospective and larger sample size studies are needed, in order to validate these results and to compare these markers with other diagnostic test to confirm their diagnostic usefulness.

In conclusion, the blood-cell-based inflammation markers could be used as an additional tool to predict the CRC prognosis and also to improve the early diagnosis in combination with other screening strategies.

## Data Availability Statement

The original contributions presented in the study are included in the article/Supplementary Material, further inquiries can be directed to the corresponding author/s.

## Ethics Statement

The studies involving human participants were reviewed and approved by Regional Ethical Committee of Aragón (CEICA). Written informed consent for participation was not required for this study in accordance with the national legislation and the institutional requirements.

## Author Contributions

MH-A collected data, analyzed data, and drafted the manuscript. RV collected and analyzed data. AL analyzed data and revised the manuscript. PC-L analyzed data, performed all statistical analysis, and drafted the manuscript. EP designed the study, analyzed data, and drafted the manuscript. All authors contributed to manuscript revision, read, and approved the submitted version.

## Funding

This research was funded by Instituto de Salud Carlos III and co-funded by European Union (ESF, Investing in your future): PI17/02171 (to EP), Diputación General de Aragón (Digestive Pathology Group B25_17R), and the Centro de Investigación Biomédica en Red en Enfermedades Hepáticas y Digestivas (CIBERehd).

## Conflict of Interest

The authors declare that the research was conducted in the absence of any commercial or financial relationships that could be construed as a potential conflict of interest.

## Publisher's Note

All claims expressed in this article are solely those of the authors and do not necessarily represent those of their affiliated organizations, or those of the publisher, the editors and the reviewers. Any product that may be evaluated in this article, or claim that may be made by its manufacturer, is not guaranteed or endorsed by the publisher.
