# Supplementary material for: Blood-Cell-Based Inflammatory Markers as a Useful Tool for Early Diagnosis in Colorectal Cancer
Source: Front Med (Lausanne). 2022 Jun 20;9:843074. doi: 10.3389/fmed.2022.843074 (PMC9252519; doi:10.3389/fmed.2022.843074)
Supplement: Supplementary file 1 [file Table_1.DOCX]

**Supplementary table 1.** Median, interquartile range (IQR) and association of the inflammatory markers according to tumor invasion (T), lymph nodes (N) and metastases (M).

|  | **NLR** median (IQR) | **PLR** median (IQR) | **SII** median (IQR) | **NP/LHb** median (IQR) |
| --- | --- | --- | --- | --- |
| **T (tumor invasion)** | | | | |
| **T1** | 1.55 (1.23-2.31) | 100 (83.64-112.34) | 400 (258.58-433.67) | 27.56 (16.59-35.06) |
| **T2** | 2.11 (1.40-4.06) | 130 (96.15-180) | 588.65 (348-952.94) | 44.65 (26.39-71.36) |
| **T3** | 2.89 (1.85-4.59) | 141 (98.79-195.16) | 643.19 (393.08-1332.03) | 52.78 /28.29-114.47) |
| **T4** | 2.63 (1.35-4.10) | 132 (92.77-179.50) | 814 (341.85-1146.83) | 62.58 (29.71-91.51) |
| **Tx** | 3.44 (2.42-5.63) | 167.53 (69.75-239.37) | 1029.05 (321.21-1633.39) | 74.31 (24.54-164.98) |
| *p-value* | ***0.003*** | ***0.047*** | ***0.007*** | ***0.011*** |
| **N (lymph nodes)** | | | | |
| **N0** | 2.31 (1.56-4.12) | 120.43 (92.96-184) | 540 (341.56-1122.73) | 44.99 (26.90-96.88) |
| **N1** | 2.67 (1.52-3.95) | 133.68 (95.98-172.03) | 603.82 (375.10-890.29) | 44.31 (27.11-70.93) |
| **N2** | 2.98 (2.30-4.71) | 150.63 (123.91-219.48) | 808.26 (490.57-1407.71) | 64.70 (37.11-128.89) |
| **Nx** | 3.30 (1.83-5.36) | 158.82 (69.99-229.58) | 1000.60 (294.31-1543.92) | 68.06 (21.94-150.50) |
| *p-value* | *0.178* | *0.140* | *0.073* | *0.139* |
| **M (metastases)** | | | | |
| M0 | 2.48 (1.67-4.17) | 127.58 (94.43-180.41) | 596.20 (346.35-987.75) | 45.28 (27.33-84.71) |
| M1 | 2.63 (1.77-4.17) | 157.30 (97.33-216.09) | 814 (424.60-1208.50) | 66.46 (33.41-107.24) |
| *p-value* | *0.493* | *0.202* | *0.093* | *0.084* |
| **Abbreviations**: NLR: neutrophil/lymphocyte ratio, PLR: platelet/lymphocyte ratio, SII: systemic immune-inflammation index, NP/LHb: neutrophils x platelets/lymphocytes x hemoglobin ratio, IQR: interquartile range, TNM stage: tumor-nodes-metastases stage. | | | | |

**Supplementary table 2.** Median, interquartile range (IQR) and association of the inflammatory markers according to symptoms at diagnosis.

|  | **NLR** median (IQR) | **PLR** median (IQR) | **SII** median (IQR) | **NP/LHb** median (IQR) |
| --- | --- | --- | --- | --- |
| **Symptoms at diagnosis** | | | | |
| Rectal bleeding (n=37) | 2.37 (1.28-3.75) | 124.28 (94.51-171.32) | 503.68 (321.53-979.04) | 37.142 (25.86-78.56) |
| Change in bowel habit (n=33) | 2.65 (2.03-4.64) | 149.25 (97.07-240.53) | 761.80 (420.78-1328.55) | 61.57 (28.18-99.20) |
| Anemia (n=33) | 2.64 (2.64-3.82) | 128.66 (96.71-179.33) | 583.77 (446.32-718.89) | 52.33 (36.37-72.25) |
| Abdominal pain (n=28) | 3 (2.04-6.13) | 145.98 (108.05-229.38) | 897.82 (460.18-1999) | 66.54 (37.16-170.77) |
| Constitutional syndrome (n=15) | 3.69 (2.50-7.42) | 190 (122.85-247.27) | 1163.07 (374-1722.85) | 113.99 (42.50-179.46) |
| Obstruction (n=14) | 5.48 (4.10-9.89) | 174.62 (136.29-225) | 1457.25 (900.87-2172) | 106.78 (65.76-180.60) |
| *p-value* | ***<0.001*** | ***<0.001*** | ***<0.001*** | ***<0.001*** |
| **Abbreviations**: NLR: neutrophil/lymphocyte ratio, PLR: platelet/lymphocyte ratio, SII: systemic immune-inflammation index, NP/LHb: neutrophils x platelets/lymphocytes x hemoglobin ratio, IQR: interquartile range, TNM stage: tumor-nodes-metastases stage. | | | | |

**Supplementary table 3.** Area under the curve (AUC) with confidence interval (CI) 95% of the inflammatory markers based on CRC diagnosis as the outcome variable in the validation cohort (n=46)

|  | AUC | 95% CI |
| --- | --- | --- |
| NLR | 0.78 | 0.64-0.91 |
| PLR | 0.80 | 0.68-0.93 |
| SII | 0.80 | 0.68-0.93 |
| NP/LHb | 0.84 | 0.73-0.95 |
| **Abbreviations**: NLR: neutrophil/lymphocyte ratio, PLR: platelet/lymphocyte ratio, SII: systemic immune-inflammation index, NP/LHb: neutrophils x platelets/lymphocytes x hemoglobin ratio, | | |

**Supplementary table 4.** Optimal cut-off values to discriminate CRC and other diagnostic parameters of the inflammatory markers in the validation cohort.

| **Markers** | **Cut-off** | **TP** | **FP** | **FN** | **TN** | **Se (%)** | **Sp (%)** | **PPV (%)** | **NPV (%)** | **OR (95% CI)** |
| --- | --- | --- | --- | --- | --- | --- | --- | --- | --- | --- |
| **NLR** | 2.28 | 15 | 6 | 8 | 17 | 65.22 | 73.91 | 71.43 | 68.00 | 5.31 (1.50-18.84) |
| **PLR** | 122.40 | 16 | 5 | 7 | 18 | 69.57 | 78.26 | 76.19 | 72.00 | 8.23 (2.18-31.13) |
| **SII** | 616.50 | 12 | 5 | 11 | 18 | 52.17 | 78.26 | 70.59 | 62.07 | 3.93 (1.09-14.20) |
| **NP/LHb** | 43.90 | 12 | 4 | 11 | 19 | 47.83 | 82.61 | 75.00 | 63.33 | 5.18 (1.34-20.06) |
| **Abbreviations**: NLR: neutrophil/lymphocyte ratio, PLR: platelet/lymphocyte ratio, SII: systemic immune-inflammation index, NP/LHb: neutrophils x platelets/lymphocytes x hemoglobin ratio, TP: true positive, FP: false positive, FN: false negative, TN: true negative, Se: sensitivity, Sp: specificity, PPV: positive predictive value, NPV: negative predictive value, OR: odds ratio, CI: confidence interval. | | | | | | | | | | |
